# *Osteomeles schwerinae* Extract Prevents Diabetes-Induced Renal Injury in Spontaneously Diabetic Torii Rats

**DOI:** 10.1155/2018/6824215

**Published:** 2018-04-24

**Authors:** Eunjin Sohn, Junghyun Kim, Chan-Sik Kim, Kyuhyung Jo, Jin Sook Kim

**Affiliations:** ^1^Korean Medicine Convergence Research Division, Korea Institute of Oriental Medicine, Daejeon 34054, Republic of Korea; ^2^Department of Oral Pathology, School of Dentistry, Chonbuk National University, Jeonju 54896, Republic of Korea

## Abstract

Mesangial cell proliferation contributes to the development of glomerulosclerosis in diabetic nephropathy. This study was aimed at determining whether* Osteomeles schwerinae* (OSSC) extract can ameliorate renal damage in Spontaneously Diabetic Torii (SDT) rats. OSSC extract (100 and 250 mg/kg/day) was administered to the SDT rats through oral gavage for 17 weeks. At the end of the experiment, glucose, HbA1c, and albuminuria were measured. In addition, the levels of mesangial proliferation-related proteins were determined by western blotting and immunohistochemistry. Our results show that albuminuria, accumulation of the extracellular matrix (ECM), and renal expansion were markedly restored by OSSC extract administration. The OSSC treatment also inhibited *α*-smooth muscle actin and transforming growth factor-*β*1 protein expression. In addition, OSSC and its bioactive compounds hyperoside and quercitrin inhibited the platelet-derived growth factor-BB (PDGF-BB)/platelet-derived growth factor-B receptor (PDGFR-*β*) ligand binding in an* in vitro* assay. Taken together, these results indicate that OSSC inhibits ECM accumulation and mesangial proliferation of the glomeruli in SDT rats through inhibition of the interaction between PDGF-BB and PDGFR-*β*. OSSC has ameliorating effects on the initiation and progression of diabetes complications and can be used for the treatment of early diabetic renal dysfunction.

## 1. Introduction

Diabetic nephropathy is a serious complication in patients with diabetes. This disease is characterized by histological alterations in the renal tissue including thickening of the glomerular basement membrane and mesangial matrix expansion, resulting in the development of glomerulosclerosis [1, 2]. Increased mesangial cell proliferation, as well as accumulation of extracellular matrix (ECM) components including collagen in the glomeruli, is one of the characteristic pathologic features in the early stages of diabetic nephropathy [3]. Platelet-derived growth factor (PDGF) facilitates various cellular actions such as cell proliferation and ECM generation in mesangial cells via interactions with its specific receptor [4]. Moreover, upregulation of PDGF-B chain (PDGF-BB) and PDGF-B receptor (PDGFR-*β*) in mesangial cells occurs in parallel with their proliferation and overproduction of ECM [5, 6].

Transforming growth factor-beta1 (TGF-ß1) is a multifunctional cytokine that plays an important role in the regulation of cell proliferation and apoptosis, as well as being a key mediator of diabetic nephropathy that is associated with increased levels of ECM proteins, such as *α*-smooth muscle actin (*α*-SMA) and collagen, in renal glomeruli [7]. Increasing evidence shows that TGF-ß1 is key pathogenic factor in the development of renal fibrosis in diabetic nephropathy [8].

*Osteomeles schwerinae* C. K. Schneid. (Rosaceae) (OSSC) is an indigenous plant in Asian countries including China. It has been used as a medicinal plant for several diseases, such as diarrhea, arthritis, sore throat, and furuncles, in traditional Chinese herbal medicine [9]. Recently, we demonstrated that OSSC has an inhibitory effect on the formation of advanced glycation end products (AGEs), as well as a preventive effect against the development of retinal pathogenic angiogenesis, in an animal model of oxygen-induced retinopathy [10]. OSSC has two bioactive compounds, hyperoside and quercitrin, which have been isolated from its ethanol extract that demonstrated an inhibitory activity on rat lens aldose reductase [11]. Furthermore, these bioactive compounds had inhibitory effects on oxidative stress induced by AGE-modified bovine serum albumin (AGE-BSA)* in vitro* [12]. Despite the reported beneficial effects of OSSC, however, its effects on diabetic nephropathy have never been studied* in vivo*. Therefore, we investigated whether OSSC extract has preventive effects on diabetic nephropathy, which is associated with glomerular proliferation in Spontaneously Diabetic Torii (SDT) fatty rat, an animal model of nonobese type 2 diabetes [13].

## 2. Materials and Methods

### 2.1. Preparation of the OSSC Extract

The plant materials of OSSC were collected from Kumming, Yunnan Province, China. Air-dried twigs and leaves of OSSC (4 kg) were subjected to maceration three times using 12 L of ethanol, followed by filtration and concentration of the extract in vacuo at 40°C, in order to derive 104.16 g of the ethanol extract. A herbarium voucher specimen (no. DiAB-141) has been deposited at the Herbarium of the Korea Institute of Oriental Medicine (Daejeon, South Korea). The contents of hyperoside and quercitrin as bioactive, or marker, compounds were quantified using an HPLC analysis that is described in our previous report [12].

### 2.2. Animal Experimental Design

Age-matched Sprague-Dawley (SD) and SDT rats (specific pathogen-free male) were obtained from the CLEA Japan (Tokyo, Japan). The rats were acclimated in controlled temperature room (22 ± 2°C, in 55 ± 10% relative humidity) with a 12 h : 12 h light-dark cycle and were fed a commercial diet (5L79, PMI Nutrition International, St Louis, MO) ad libitum and given tap water freely in cages (5 rats per cage). At 24 weeks of age, the rats were randomized into the following 4 groups: nondiabetic control rats (NOR, *n* = 10), diabetic SDT rats (DM, *n* = 10), SDT rats treated with OSSC at 100 mg/kg (OSSC-100, *n* = 10), and SDT rats treated with OSSC at 250 mg/kg (OSSC-250, *n* = 10). OSSC was dissolved in distilled water and given orally once a day for 17 weeks. No evidence of systemic adverse effects was founded in experimental group. Twenty-four-hour urine was collected in metabolic cages at the end of the study. At the end of the experimental period, the rats were sacrificed deeply anesthetized with CO_2_ asphyxiation after fasting for 12 h followed by collection of blood and kidney samples. All animal care procedures were approved by the Institutional Animal Care and Use Committee of Korea Institute of Oriental Medicine (Deajeon, Korea) (IACUC Approval number HH109037).

### 2.3. Measurement of Blood and Urine Samples

Blood samples were obtained at the time of sacrifice and blood glucose was measured with an automated biochemistry analyzer (HITACHI 917, Japan). Albumin levels in urine were measured by enzyme-linked immunoabsorbent assay using an anti-rat albumin antibody as previously described [14].

### 2.4. Morphological Analysis

Periodic acid–Schiff (PAS) and Masson's modified trichrome stain were performed for assessing glomerulosclerosis. Glomerular volume was measured using image J software (NIH, Bethesda, MD, USA), and the area of one glomerulus in a total of 30 glomeruli was determined.

### 2.5. Immunohistochemistry

Kidney sections were dewaxed and rehydrated with xylene and graded ethanol series. The preparations were incubated with 3% H_2_O_2_ to inhibit endogenous peroxidase activity followed by boiling in 10 mM citrate buffer (pH 6.0) for 10 min in a microwave. These sections were washed with PBS buffer and blocked with 1% animal free serum for 30 min at 37°C. The sections were subsequently incubated with anti-TGF-ß1 and anti-*α*-SMA (Santa Cruz, CA, USA) primary antibodies for 2 h at 37°C for immunohistochemistry. The sections were incubated with Envision dual peroxidase kit (Dako, Denmark) for 30 min at room temperature and treated with peroxidase substrate solution containing diaminobenzidine (DAB, Dako, Denmark) or aminoethyl carbazole (AEC, Vector, Burlingame, CA, USA). Stained sections were then visualized using an Olympus DP71 camera connected to Olympus microscope (Tokyo, Japan) at 400x magnification. A minimum of 15 glomerular fields (magnification 400x) per kidney were evaluated in all kidney tissues and the images were recorded and analyzed using Image J software (Java-based image processing program, NIH).

### 2.6. Western Blot Analysis

Renal cortical tissue was homogenized in 0.1 M Tris-based homogenization buffer (pH 7.4). Protein lysates (25 *μ*g) were separated by sodium dodecyl sulfate-polyacrylamide gel electrophoresis (SDS-PAGE). Proteins on the gel were next transferred onto a PVDF membrane (BioRad, CA, USA), which was incubated with antibodies subsequently.

### 2.7. Ligand Receptor Binding Inhibition In Vitro Assay

PDGF-BB and PDGFR-*β* receptor ligand binding was examined using a sandwich ELISA assay according to a previously reported protocol [15]. Briefly, recombinant human PDGF-BB (R&D systems, MN, USA) was coated onto a 96-well plate. Next, 50 *μ*l of PDGFR-*β*/FC chimera labeled with peroxidase (Dojindo, Kumanoto, Japan) and 50 *μ*l of a serial dilution of the OSSC extract and its two bioactive compounds were added on the microplate and incubated for 1 h at 37°C. The binding of PDGF-BB to PDGFR-*β* was detected using a H_2_O_2_ substrate containing a tetramethylbenzidine (TMB) chromogen. Binding levels were measured as the percentage decrease in optical density (OD = 450 nm). IC_50_ concentration (*μ*g/mL) was calculated as the 50% inhibition of PDGF-BB to PDGFR-*β* binding.

### 2.8. Statistical Analysis

Data are expressed as mean ± SEM. values. Unpaired Student's *t*-tests were used to compare two groups using GraphPad Prism software (Graph Pad, San Diego, CA, USA). Differences of *p* < 0.05 were considered statistically significant.

## 3. Results

### 3.1. Body Weight, Food Intake, Blood Glucose, HbA1c, and Albuminuria

Table 1 shows body weight, blood glucose, and renal function data after 17 weeks of OSSC treatment. Food intake, blood glucose, HbA1c levels, and albuminuria were significantly higher in SDT rats than in the normal rats, whereas body weight was markedly lower in the diabetic rats than in the normal rats. Albuminuria, a marker of renal function, was markedly lower in the SDT rats treated with OSSC without a concomitant reduction in blood glucose and HbA1c levels. In addition, body weight loss was ameliorated by OSSC treatment in the SDT rats. However, no difference of the food intake was noted between all diabetic groups.

### 3.2. Morphological Analysis

**Figure 1:**
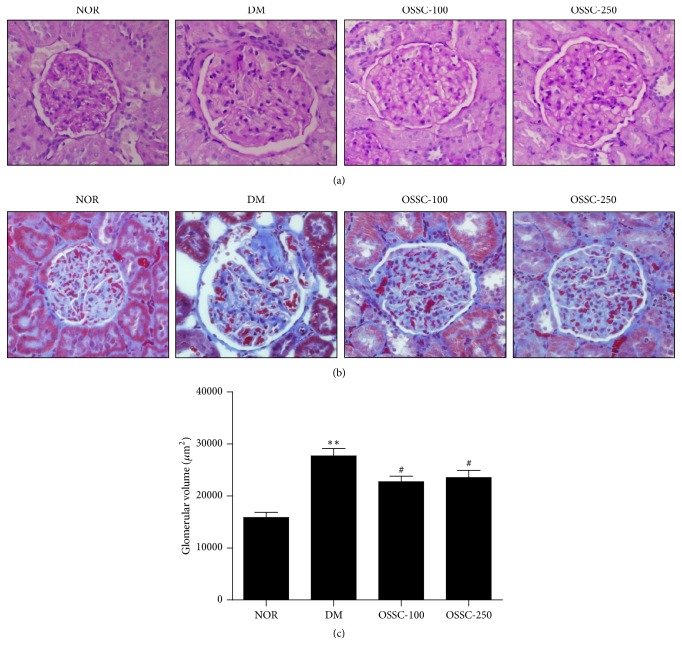
Renal morphology and glomerular volume. Periodic acid–Schiff (PAS) (a) and Masson's modified trichrome stain. 400x magnification. (b). Glomerular volume. (c) Experimental groups: NOR, normal rat; DM, SDT rat; OSSC-100, DM treated with OSSC (100 mg/kg); OSSC-250, DM treated with OSSC (250 mg/kg). All data are expressed as mean ± SEM values (*n* = 10). ^*∗∗*^*p* < 0.001 versus NOR; ^#^*p* < 0.01 versus DM.

Histological analysis showed that renal glomeruli in the SDT rats exhibited mesangial proliferation and matrix expansion, as well as the accumulation of collagen (Figures 1(a) and 1(b)). Glomerular volume and mesangial proliferation were significantly higher in the SDT rats than in the normal rats. However, OSSC treatment was associated with a marked reduction in matrix expansion, collagen accumulation, and glomerular volume in the SDT rats (Figures 1(a), 1(b), and 1(c)).

### 3.3. Effects of OSSC Treatment on the Expression of Renal *α*-SMA and TGF-ß1

**Figure 2:**
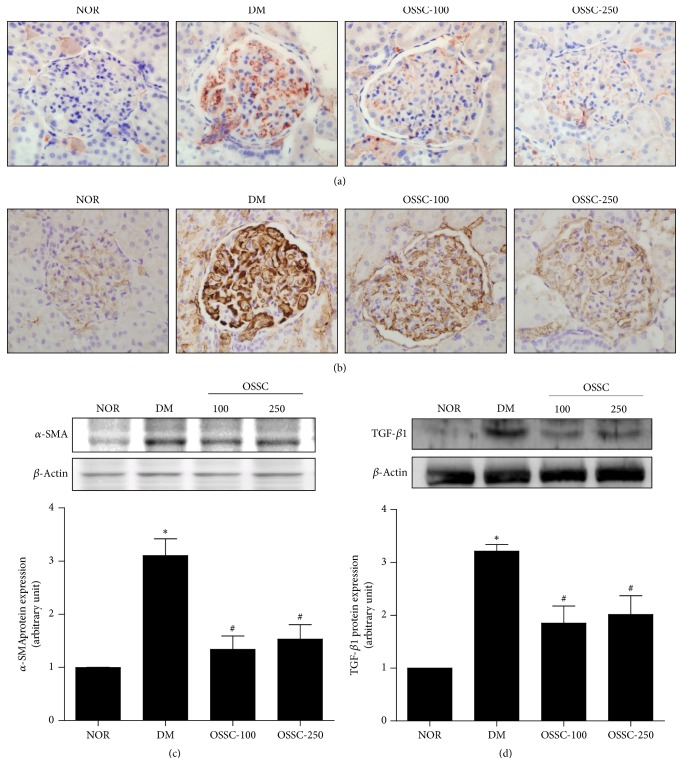
Effects of OSSC on the level of *α*-SMA and TGF-ß1 in renal tissue. Immunohistochemical staining of *α*-SMA (a) and TGF-ß1 (b). 400x magnification. *α*-SMA (c) and TGF-ß1 (d) expression levels in the renal tissue in each group. Experimental groups: NOR, normal rat; DM, SDT rat; OSSC-100, DM treated with OSSC (100 mg/kg); OSSC-250, DM treated with OSSC (250 mg/kg). All data are expressed as mean ± SEM values (*n* = 10). ^*∗*^*p* < 0.05 versus NOR; ^#^*p* < 0.01 versus DM.

We examined the expression of *α*-SMA and TGF-ß1 in the renal cortex. As shown in Figure 2, glomeruli of the SDT rats showed a significant increase in the expression of *α*-SMA and TGF-ß1 proteins as revealed by cytoplasmic immunostaining (Figures 2(a) and 2(b)). OSSC treatment, however, was associated with marked reduction in the expression of *α*-SMA and TGF-ß1 in the renal glomeruli of SDT rats compared to that in the normal rats. Immunohistochemical and western blot analysis of *α*-SMA and TGF-ß1 showed an increase in the expression of these proteins in SDT rats compared with that in normal rats, which reduced with OSSC treatment (Figure 2).

### 3.4. Effect of OSSC Treatment on the Expression of PDGF-BB and PDGFR-ß

**Figure 3:**
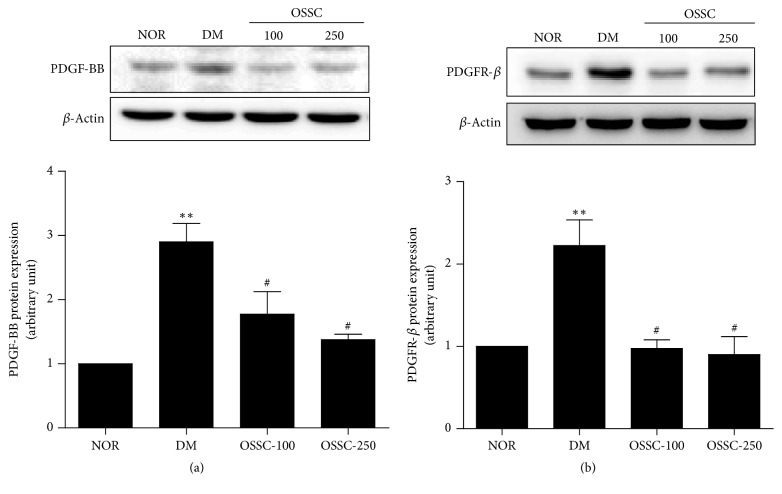
Effects of OSSC on the level of PDGF-BB and PDGFR-ß in renal tissue. Western blot analysis of PDGF-BB (a) and PDGFR-ß (b) expression in renal tissue in each group. Experimental groups: NOR, normal rat; DM, SDT rat; OSSC-100, DM treated with OSSC (100 mg/kg); OSSC-250, DM treated with OSSC (250 mg/kg). All data are expressed as mean ± SEM values (*n* = 10). ^*∗∗*^*p* < 0.001 versus NOR; ^#^*p* < 0.01 versus DM.

PDGF plays an important role in the production of ECM proteins in the renal glomeruli during early lesions of diabetic nephropathy [16]. To investigate whether OSSC treatment could decrease the expression of PDGF-BB and PDGFR-ß proteins in the renal cortex of SDT rats, western blotting was performed using antibodies for PDGF-BB and PDGFR-ß receptor. PDGF-BB and PDGFR-ß protein levels significantly increased in the renal cortex of the SDT rats compared to those in the normal rats. However, OSSC treatment was associated with marked reduction in the expression of PDGF-BB and its receptor PDGFR-ß in the SDT rats (Figures 3(a) and 3(b)).

### 3.5. Inhibitory Effect of OSSC on PDGF-BB Ligand Binding to PDGFR-ß

OSSC contains two major bioactive compounds, hyperoside and quercitrin [11]. It showed a marked inhibitory effect (1.46 ± 0.36 *μ*g/ml) on the binding of the PDGF-BB ligand to its receptor. In addition, both hyperoside and quercitrin also exhibited inhibitory activities (5.46 ± 0.11 and 142.86 ± 14.50 *μ*g/ml, resp.) on the binding of the PDGF-BB ligand to its receptor (Table 2).

## 4. Discussion

This study demonstrated that OSSC treatment markedly ameliorated albuminuria, body weight loss, mesangial expansion, and collagen accumulation in the renal glomeruli of diabetic rats. OSSC treatment also exhibited inhibitory effects on the binding of PDGF-BB to its receptor, PDGFR-ß, and further reduced their protein expression (i.e., of both PDGF-BB and PDGFR-ß) in the renal cortex of the SDT rats. In addition, OSSC treatment was associated with the reduction of *α*-SMA and TGF-ß1 protein expression in the SDT rats. These results suggest that the renoprotective effects of OSSC are likely mediated by the inhibition of the binding of PDGF-BB to PDGFR-ß, resulting in the prevention of renal glomerular proliferation and renal damage in the SDT rats.

The SDT rat is an animal model of nonobese type 2 diabetes, which exhibits hyperglycemia and albuminuria, characteristic features of diabetic nephropathy [17, 18]. This animal model resembles human diabetic nephropathy in terms of both morphological and functional kidney damage [19]. Based on the results of a previous study [19], we believe we have used an appropriate animal model for an investigation related to diabetic nephropathy. Hyperglycemia is a strong risk factor for the development of diabetic nephropathy. Mesangial cell proliferation and collagen accumulation in the matrix, as well as thickening of the basement membrane of the renal tissue, result in the leakage of albumin or protein in the urine. Albuminuria is a main pathologic feature of diabetic nephropathy including many primary glomerular diseases [20]. This study demonstrated that albuminuria and glomerular matrix expansion were significantly higher in the SDT rats. However, our study further revealed that OSSC treatment ameliorated diabetes-induced albuminuria and glomerular matrix expansion in the SDT rats.

OSSC treatment did not affect blood glucose and HbA1c levels in the SDT rats. This is consistent with the well-known antidiabetic drugs, such as metformin and dipeptidyl peptidase IV inhibitors, having no effect on blood glucose levels in the diabetic animal models. Indeed, these studies have been used for studying the mechanisms behind the beneficial effects of antidiabetic drugs on diabetic nephropathy, independent of their blood glucose lowering effects [21–24]. Therefore, this study demonstrated that OSSC treatment has renoprotective effect on renal dysfunction in the SDT rats without blood glucose lowering effect. The OSSC treatment did not show the dose-dependent response. One of the possible reasons for this may be that the OSSC possess a strong antidiabetic complication activity independent of glycemic control. The concentration of active principle at the given 100 mg/kg dose might be capable of controlling at reach sufficient concentration of renal function recovery in SDT rats without dose-dependent effect in kidney. Thus, OSSC treatment was reduced diabetes-induced renal dysfunctions by ameliorating albuminuria and glomerular matrix expansion in SDT rat.

Our study also showed that the OSSC treatment has preventive effects against mesangial expansion and proliferation in the renal glomeruli of SDT rats by inhibiting the binding of PDGF-BB to its receptor, PDGFR-ß. Abnormal proliferation of mesangial cells and enhanced ECM deposition are the characteristic risk factors for the development of diabetic nephropathy that further leads to the development of chronic kidney disease [25]. PDGF was originally purified as a polypeptide from human platelets with potent mitogen activities for fibroblasts, mesangial cells, and smooth muscle cells [1]. Moreover, multiple studies have indicated that PDGF may contribute to the initiation and progression of fibrosis during diabetic nephropathy [26, 27]. PDGF-BB, one of the isoforms of PDGF, binds to its receptor PDGFR-ß, resulting in an increase in the glomerular matrix thereby inducing mesangial expansion and proliferation [27, 28]. Genetic deletion of PDGFR-ß has been shown to reduce renal injury, in particular mesangial expansion, in murine models of diabetic nephropathy [29–31]. It is likely that PDGFR-ß is involved in the development of diabetic nephropathy and diabetes even beyond its specific role in mesangial expansion. In addition, TGF-ß1 is known to be involved in the regulation of ECM synthesis and has strong fibrogenic effects that result from its dual impact on the stimulation of matrix synthesis as well as blockade of matrix degradation during renal disease [32]. Fraser et al. reported that PDGF-BB can promote fibrosis during diabetic nephropathy via upregulation of TGF-ß1, which can be induced by PDGF-BB in the tubular cells [33, 34]. Similarly, *α*-SMA, one of the ECM proteins, has been regarded to be a predictor of diabetic nephropathy and is highly upregulated in the animal models of diabetic nephropathy [32, 35, 36]. Our study showed that OSSC and its bioactive compounds, hyperoside and quercitrin, inhibited the PDGF-BB/PDGFR-ß ligand binding in an* in vitro* assay. Based on these findings, we suggest that OSSC treatment potentially ameliorated diabetic nephropathy in the SDT rats through its anti-PDGF or PDGFR activities. Taken together, these results indicate that OSSC inhibits ECM accumulation and mesangial proliferation in the renal glomeruli of diabetic rats through inhibition of the interaction between PDGF-BB and PDGFR-ß.

In conclusion, this is the first study to provide evidence that OSSC treatment can inhibit experimental diabetic nephropathy. In addition, our* in vitro* experiment showed that OSSC and its bioactive compounds, hyperoside and quercitrin, also inhibited the interaction between PDGF-BB and PDGFR-ß. Further studies are required to determine the feasibility of using OSSC for the treatment of patients with diabetic nephropathy.

## Figures and Tables

**Table 1:** Parameters of experimental rats.

	NOR	DM	OSSC-100	OSSC-250
Body weight (g)	713.9 ± 29.7	375.5 ± 52.4^1^	415.0 ± 61.5	420.9 ± 33.4^2^
Food intake (g/day)	24.5 ± 2.2	43.6 ± 4.3^1^	47.5 ± 8.1	40.3 ± 12.7
Blood glucose (mg/dl)	144.1 ± 21.0	419.2 ± 21.1^1^	393.4 ± 47.7	391.5 ± 52.2
HbA1C (%)	3.49 ± 0.07	9.13 ± 0.37^1^	9.14 ± 0.31	8.74 ± 0.48
Albuminuria (mg/day)	1.55 ± 0.21	12.75 ± 2.50^1^	8.49 ± 0.88^2^	7.59 ± 2.07^2^

NOR, normal rat; DM, SDT rat; OSSC-100, DM treated with OSSC (100 mg/kg); OSSC-250, DM treated with OSSC (250 mg/kg). All data are expressed as the mean ± SEM (*n* = 10). ^1^*p* < 0.05 versus NOR; ^2^*p* < 0.05 versus DM.

**Table 2:** PDGF-BB and ligand binding inhibition.

Compound name	Concentration (ug/ml)	% inhibition	IC_50_ (ug/ml)
OSSC	0.5	56.68 ± 7.25	1.46 ± 0.36
	1.0	52.06 ± 1.74	
	2.5	45.79 ± 1.55	

Hyperoside	2.5	63.08 ± 4.43	5.46 ± 0.11
	5	52.99 ± 6.98	
	10	29.31 ± 2.37	

Quercitrin	50	68.09 ± 16.55	142.86 ± 14.50
	100	49.73 ± 3.32	
	200	4.91 ± 2.84	

Inhibitory activity was expressed as the mean ± SEM values from triplicate experiments. The IC_50_ value was calculated from the dose inhibition curve.

## Data Availability

The datasets used and/or analyzed during the current study are available from the corresponding author on reasonable request.
